# The Fabrication and Characterization of Ni/4H-SiC Schottky Diode Radiation Detectors with a Sensitive Area of up to 4 cm^2^

**DOI:** 10.3390/s17102334

**Published:** 2017-10-13

**Authors:** Lin-Yue Liu, Ling Wang, Peng Jin, Jin-Liang Liu, Xian-Peng Zhang, Liang Chen, Jiang-Fu Zhang, Xiao-Ping Ouyang, Ao Liu, Run-Hua Huang, Song Bai

**Affiliations:** 1 School of Nuclear Science and Technology, Xi’an Jiaotong University, No. 28, Xianning West Road, Xi’an 710049, China; liulinyue@nint.ac.cn; 2 State Key Laboratory of Intense Pulsed Radiation Simulation and Effect, Northwest Institute of Nuclear Technology, Xi’an 710024, China; jinpeng@nint.ac.cn (P.J.); liujinliang@nint.ac.cn (J.-L.L.); zhangxianpeng@nint.ac.cn (X.-P.Z.); chenliang@nint.ac.cn (L.C.); zhangjianfu@nint.ac.cn (J.-F.Z.); 3 State Key Laboratory of Wide-Bandgap Semiconductor Power Electronic Devices, Nanjing Electronic Devices Institute, No. 524 East Zhongshan Road, Nanjing 210016, China; wanglinghao122@163.com (L.W.); 15851831604@163.com (A.L.); 18626422152@163.com (R.-H.H.); 4 Shaanxi Engineering Research Center for Pulse-Neutron Source and its Application, Xijing University, Xi’an 710123, China

**Keywords:** 4H-SiC, radiation detection, large sensitive area, Schottky diode

## Abstract

Silicon carbide (SiC) detectors of an Ni/4H-SiC Schottky diode structure and with sensitive areas of 1–4 cm^2^ were fabricated using high-quality lightly doped epitaxial 4H-SiC material, and were tested in the detection of alpha particles and pulsed X-rays/UV-light. A linear energy response to alpha particles ranging from 5.157 to 5.805 MeV was obtained. The detectors were proved to have a low dark current, a good energy resolution, and a high neutron/gamma discrimination for pulsed radiation, showing the advantages in charged particle detection and neutron detection in high-temperature and high-radiation environments.

## 1. Introduction

Since the first silicon carbide (SiC) detector was developed nearly sixty years ago [1,2,3], the potentials of SiC detectors have been recognized for their better endurance to elevated temperatures and radiation-induced damage than conventional silicon or germanium detectors. Many other semiconductors have been used to fabricate detectors at the same time: CdTe, CdZnTe, GaAs, and AlInP are focused on photon detection [4,5,6]; diamond is suitable for neutron, photon, and charged particle detection and has ultra-high radiation resistance but with tiny dimension, uneven quality, and high cost [7,8]. By now, SiC detectors have been demonstrated to have a high resolution in the detection of charged particles [9,10,11,12,13,14], photons [15,16,17,18], and neutrons [19,20,21,22]. Particularly, because of their outstanding operations in applications in intense radiation fields and harsh environments, such as alpha particle monitoring and neutron detection in actinide waste-tank environments [23] and neutron and gamma-ray monitoring of spent nuclear fuel assemblies [24,25], and because the technology has matured in terms of material growth and device fabrication, they have been considered preferable substitutions for conventional silicon radiation detectors.

However, compared with commercial silicon detectors whose sensitive areas are usually in the range of 0.78–7 cm^2^, even up to 70 cm^2^ in some applications, the largest sensitive area of an SiC spectrometry detector is only 0.36 cm^2^ for a single chip [26] or 0.81 cm^2^ for a splicing device [27]. Small detectors have been studied sufficiently and show good performance in charged particle monitoring, etc., but usually they have low efficiency in radiation beams with large diameters or large radiation emission angles, and thus need more time to accumulate sufficient counts to ensure that the results meet the statistical requirements. For the detection in a large radiation field, larger detectors are required. 

Most high-quality SiC detectors are made with epitaxial SiC material. The low dark current is necessary for SiC detectors, both in spectroscopic and in current mode detection. The fabrication of large-area SiC detectors is a difficult task due to the defects in epitaxial material and micro-pipes in the SiC substrate, which will cause excessive leakage current and a reduction in breakdown voltage, thus resulting in the degradation of the response properties of SiC detectors. We fabricated a passel of SiC Schottky diode chips with a size of 1 cm × 1 cm using lightly doped 4H-SiC epitaxial material 20 μm thick, and assembled two groups of large-area SiC detectors, each with four chips in a 2 × 2 array on a PCB plate and a ceramic case. The properties of the detectors were experimentally studied, and the following results were achieved: a dark current of 15–60 nA at 600 V, an optimum energy resolution of 3.22% for alpha particles, a rise time of 9.4 ns, and a neutron/gamma discrimination of 126. 

## 2. Experimental Section

### 2.1. The Fabrication of 4H-SiC Detectors 

**Figure 1:**
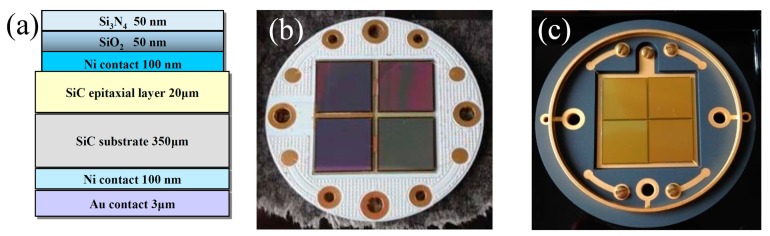
(**a**) Schematic diagram of a 4H-SiC Schottky diode detector; photograph of a 4H-SiC detector in a 2 × 2 array mounted on a multi-layer PCB plate (**b**) and in a ceramic case (**c**) with a total sensitive area of 4 cm^2^.

The high-quality lightly doped epitaxial 4H-SiC material was grown via chemical vapor deposition (CVD) on commercial 4H-SiC N+ conducting substrate wafers (Φ 10.2 cm × 350 μm, and a target nitrogen doping concentration of 10^19^ cm^−3^, supplied by TankeBlue Semiconductor Co. Ltd., Beijing, China). The epitaxial layers were 20 μm thick and with target nitrogen doping concentrations of 1–5 × 10^14^ cm^−3^. The top Schottky barrier was formed by the deposition of 100 nm nickel on epitaxial layers via thermal vacuum evaporation, and was protected by multi-layers of monox/silicon nitride (50 nm/50 nm) that covered the nickel electrode. The bottom ohmic contact was acquired by evaporation of Ni/Au and then annealing at 900 °C in nitrogen. The front contact was protected by multi-floating rings from high voltage damage. Figure 1a shows a schematic diagram of a 4H-SiC Schottky diode detector. 

Normally, the yield of an SiC detector will be limited by the concentration of the defects in the detector [26]. Detectors of a larger diameter are more likely to contain more defects in their active area, which will degrade their response properties, such as excessive leakage current. Initially, we attempted to make an area scale-up of a diode with a sensitive area up to 25 mm^2^, and following encouraging results, fabricated a passel of diode chips with 100 mm^2^ in sensitive area equivalents. We assembled two groups of detectors, each with four chips connected in parallel in a 2 × 2 array—one group on a PCB plate, the other on a ceramic case. The back electrode was connected by a welding process and the front electrode was linked by bonding with Au wires. Figure 1b,c are the pictures of the diode chips connected to a PCB plate and a ceramic shell, respectively. Each SiC detector has a sensitive volume of 20 mm × 20 mm × 20 μm and a dead layer of Ni/SiO_2_/Si_3_N_4_ (100 nm/50 nm/50 nm) without considering the dead region in the SiC near the Schottky contact.

### 2.2. Measurements

Both the forward I-V and C-V curves of the detector were measured using Agilent B1500A Power Device Analyzer/ Curve Tracer. The dark current was measured by Keithley 6517A Ampere Meter in a shielded copper box in darkness. A PS350 high voltage supply (Stanford research system Inc., Sunnyvale, CA, USA) was used to provide the reverse bias.

**Figure 2:**
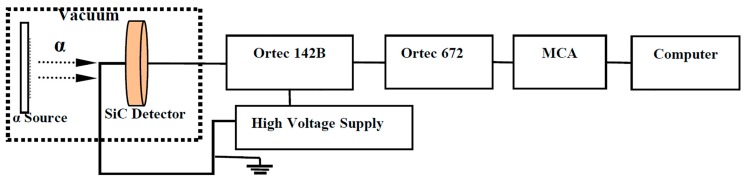
Experimental setup for alpha particle detection with the SiC detector.

The response of the SiC detectors to charged particles was studied experimentally with the alpha sources in a vacuum chamber in Nuclear Institute of Northwest Technology (NINT) in Xi’an, China. One alpha source was mixed with ^243^Am (E_α_ = 5.275 MeV, branch ratio of 87.5%) and ^244^Cm (E_α_ = 5.805 MeV, a branch ratio of 76.4%) with a radioactivity of 1.8 × 10^3^ Bq, the other was ^239^Pu (E_α_ = 5.157 MeV, branch ratio of 73.3%) with a radioactivity of 1.2 × 10^5^ Bq. Both alpha sources were prepared via the electro-deposition of oxidized isotopes on stainless-steel plates—one with a diameter of 10 mm and the other of 30 mm. As shown in Figure 2, the alpha sources were positioned concentrically with the detector’s sensitive layer, 80 mm away from the detector. The signals from the detector were amplified by an Ortec-142B Pre-Amplifier and an Ortec-672 Amplifier with a shaping time of 1 μs and a gain of 50, and were then analyzed by an Ortec multichannel analyzer (MCA) and Gamma-Vision software. The reverse bias voltages of 0–500 V were applied to the detector by the PS350 bias supply through the Ortec 142B preamplifier.

The response time of a semiconductor detector is one of the key parameters in pulsed radiation detection. It can be determined in the detection of prompt pulsed radiation from a source fast enough to be assumed as a delta (δ) source. In the experiment described here, a pulsed sub-nanosecond X-ray source and a pulsed UV laser device provided by NINT were used. The pulsed sub-nanosecond X-ray source emits a pulsed X-ray beam on average lower than 100 keV, with a rise time around 600 ps and a repetition frequency of 1 Hz. The UV laser device (EKSPLA PL2251C) emits 355 nm pulsed UV-light with a pulse-width of 30 ps and a maximum energy of 20 mJ in each shot. The response waveforms were recorded by a Tektronix 4104 Oscilloscope (bandwidth: 1 GHz; sample-rate: 4 GS/s) and a Lecroy 6100A Oscilloscope (bandwidth: 1 GHz; sample-rate: 10 GS/s) through well-shielded cables. 

## 3. Results and Discussion

### 3.1. Electric Parameters

**Figure 3:**
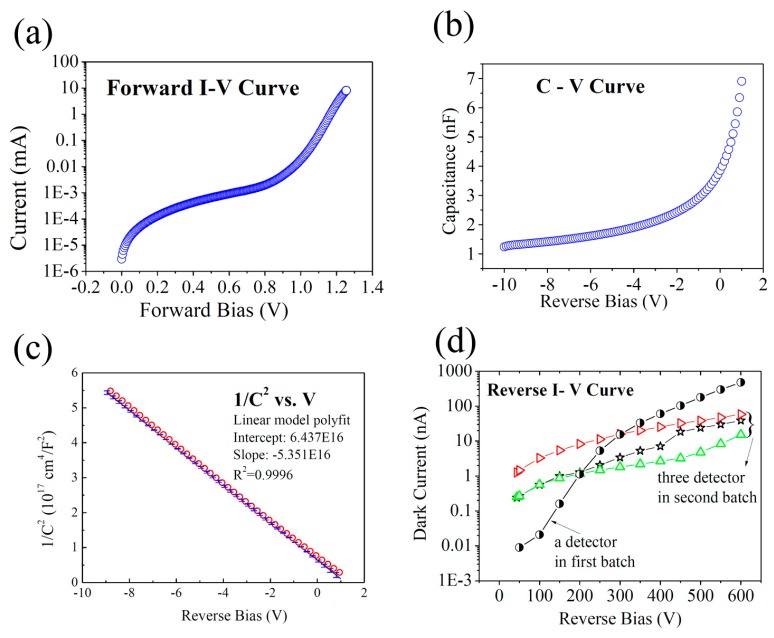
Electric parameters of the 4H-SiC diode: (**a**) Forward I-V of one chip; (**b**) C-V curve of one chip ; (**c**) 1/C^2^-V plot of one chip; (**d**) Reverse I-V (Dark current) of an SiC detector from the PCB group (half block circle in black) and three detectors from the ceramic shell group (open right-triangle in red, open star in black, and open up-triangle in green) from the four pixel structure at a reverse bias of 600 V.

The result of the forward I-V test is shown in Figure 3a. The curve exhibits a rectification character. According to the forward I-V characteristics and the Bethe equation, we find the ideality factor is 1.422 ± 0.005, which indicates the current is not just dominated by thermionic current—the diffusion current and recombination current are contributing too. [27] 

Figure 3b shows the C-V curve acquired at 1 MHz. Figure 3c is the curve of 1/C^2^ vs. V. The effective doping concentration (N_eff_) of the 4H-SiC epitaxial layer was calculated to be (2.721 ± 0.004) × 10^14^ cm^−3^ and the built-in V_bi_ potential of the Schottky contact was found to be 1.229 ± 0.007 eV. The Schottky barrier height was about 1.513 ± 0.009 eV. Figure 3d shows the dark current of an SiC detector from the PCB plate group (first batch). The dark current is 0.48 μA at a reverse bias of 600 V, which is higher than what we expected. We then made some technical optimization to the other group of the detectors, including reducing the doping concentration of the SiC epitaxial layer, selecting SiC wafers with low defect density, adjusting the annealing temperature of the bottom Ni/Au electrode, and improving the surface roughness of the SiC material near the front Ni electrode, and we then measured the dark current of the three detectors in the ceramic shell (second batch). We found that the dark current decreased to 15.2 nA, 38.8 nA, and 58.6 nA with an uncertainty within 1%, respectively, at a reverse bias of 600 V. The dark current of the second group of detectors was much lower than those of conventional silicon PIN detectors of the same dimensions (higher than 1 μA) [28].

### 3.2. Alpha-Particle Detection–Steady State Measurement

In the detection of charged particles and ion beams, once the charged particles, such as protons and alpha (α) particles, are incident on the SiC material, ionization will occur, causing the incident charged particles to lose part or all of their energy, resulting in the formation of electrons and holes (called charged carriers). The charged carriers drift in the bias field of the detector and are collected by the electrodes. Using SRIM code [29], we calculated the energy of the incident particles emitted from the ^239^Pu, and ^243^Am-^244^Cm sources after they passed through the Si_3_N_4_/SiO_2_/Ni entrance layer (dead layer) and found that all of their residual kinetic energy was lost in the active volume of the detector.

**Figure 4:**
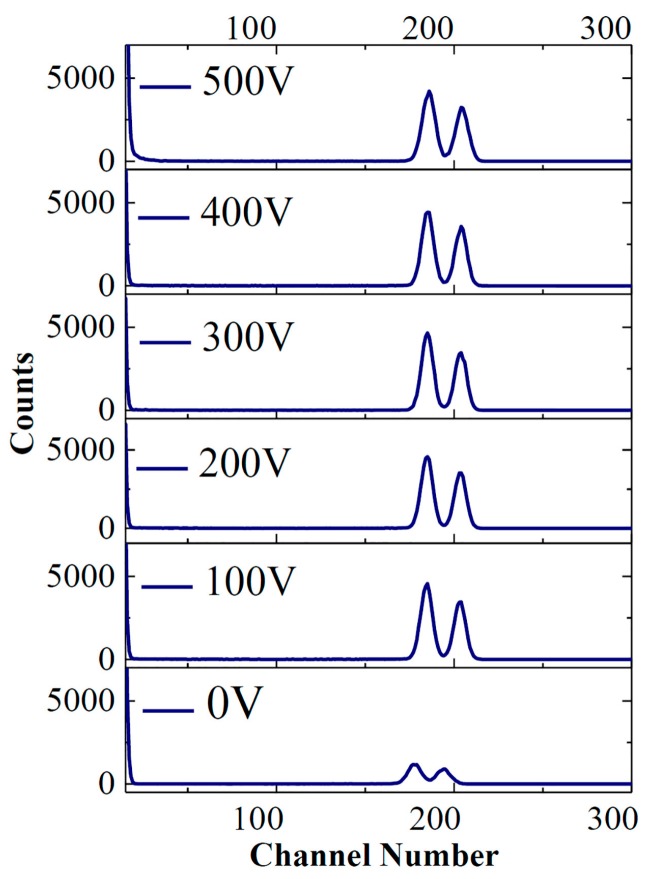
Response of the 4H-SiC detector to alpha particles emitted by ^243^Am and ^244^Cm at reverse bias voltages of 0, 100 V, 200 V, 300 V, 400 V, and 500 V.

**Figure 5:**
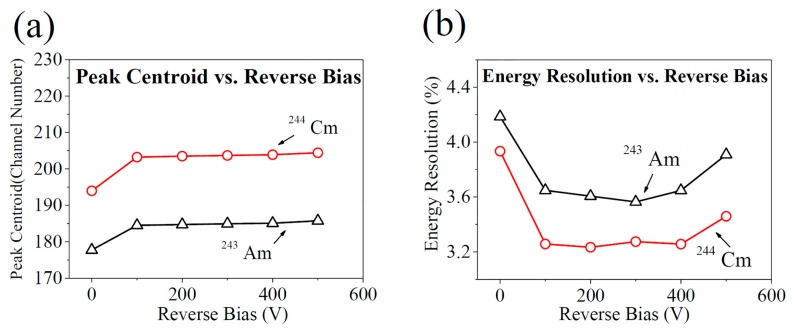
Characteristics of the pulse height spectra of the 4H-SiC detector in response to the alpha particles emitted from the ^243^Am (black open triangle) and ^244^Cm (red open circle) source (**a**) channel number of alpha peak centroid as a function of applied reverse bias voltages ranging from 0 to 500 V; (**b**) energy resolution as a function of reverse bias voltage.

Figure 4 shows the response spectra of the detector to the alpha particles emitted by the source of ^243^Am-^244^Cm at the reverse bias voltages of 0, 100 V, 200 V, 300 V, 400 V, and 500 V. It is worth noting that the detector attained similar alpha response spectra and worked stably at reverse bias voltages no less than 100 V, but measurable numbers lost amounts of incident events induced by alpha particles at a reverse bias of 0. Figure 5a gives the peak centroid as a function of reverse bias voltage. The peak centroid in the spectrum at 0 V (without reverse bias) is about 5% lower than those in the other spectra at the reverse bias voltages of 100–500 V. Fitting the peaks obtained above by the Gaussian function, we got the full width at half maximums (FWHMs). By dividing the FWHM by the peak centroid, we got the energy resolution as a function of reverse bias (Figure 5b). The best energy resolution is at 200 V and 300 V. The rise of energy resolution at reverse bias voltages above 400 V can be due to the increase in the SiC detector’s white noise, which could increase the detector’s electronic noise and broaden the alpha peaks.

**Figure 6:**
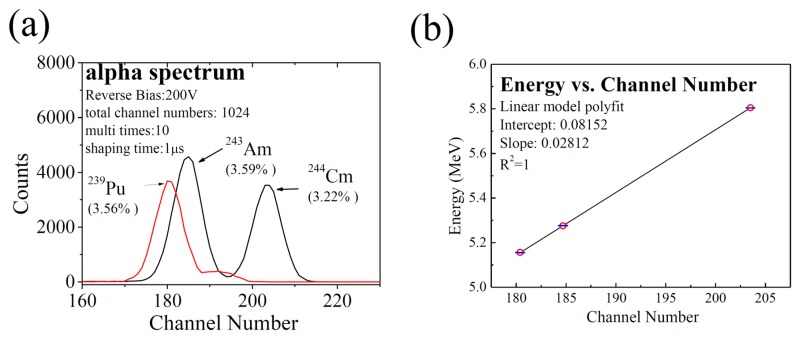
Response spectra of the 4H-SiC detector to the alpha particles from the sources of ^239^Pu,^243^Am, and ^244^Cm at a reverse bias of 200 V: (**a**) alpha counts as a function of channel number; (**b**) linear fitting of alpha particles’ energy vs. channel number of peak centroid.

The response spectra to ^239^Pu, ^243^Am, and ^244^Cm alpha particles at a reverse bias of 200 V is shown in Figure 6a, which is expressed by the counts of the alpha particles as a function of channel number. Three sharp alpha-particle peaks can be clearly observed. The energy of alpha particles as a function of observed peak centroid’s channel number is shown in Figure 6b. The energy and channel number of the centroid of the three peaks are linearly correlated with a correlation factor (R^2^), very close to 1. The average deviation is 1.33 keV over the range of 5.157 MeV and 5.805 MeV. 

Gaussian fitting was made with the peaks acquired in Figure 6a, and the FWHMs of the three peaks were attained: 183.5 keV for ^239^Pu, 190.2 keV for ^243^Am, and 187.7 keV for ^244^Cm. Many factors may contribute to the results: the statistical broadening (about 5.9 keV for ^239^Pu, 6.0 keV for ^243^Am, and 6.3 keV for ^244^Cm) [14,30], the energy straggling of the dead layer (about 11 keV) [31], the electronic noise (about 10 keV), etc. Excluding the influence of statistical broadening, the dead layer’s straggling, and the electronic noise, we attained the inherent FWHMs of 182.8 keV for ^239^Pu, 189.5 keV for ^243^Am, and 187.0 keV for ^244^Cm, as well as an optimum energy resolution of about 3.22% at a reverse bias voltage of 200 V. 

### 3.3. Response Time—Pulsed Radiation Detection

**Figure 7:**
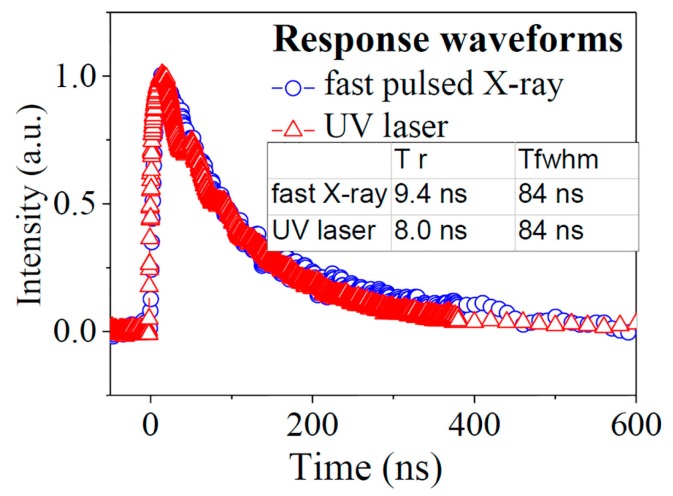
Response waveforms of a 4H-SiC detector with a dimension of 20 mm × 20 mm × 20 μm at a reverse bias of 400 V: response waveform to fast pulsed X-ray (blue open circle) and to ultra UV-light (red open triangle).

The response waveforms of the SiC detector to the pulsed X-rays and UV-light are shown in Figure 7. If the pulse height of the detector for the two pulsed sources were normalized, the response waveforms would be little different. The rise time for X-ray and UV-light waveforms is 9.4 ns and 8.0 ns, while the FWHM for X-ray and UV-light waveforms are both 84 ns. The difference can be attributed to the fact that the excitation of charged carriers occurred in the whole sensitive volume for X-rays, while for UV-light, it only occurred in the thin layer of sensitive volume near the incident surface. 

According to Dikinson’s theory [32], the rise time and the FWHM of an SiC detector can be improved significantly by increasing the detector’s sensitive thickness. This effectively achieves a faster time response. 

### 3.4. Neutron/Gamma Discrimination 

One of the most important applications of SiC detectors is neutron detection. SiC detectors have a relatively high radiation resistance. It was reported that the dose threshold for the onset of damage in an SiC film detector could be three orders of magnitude higher than that in a silicon PIN detector. [33] Besides, SiC detectors have a high neutron/gamma discrimination (n/γ discrimination), which makes SiC detectors good tools for neutron detection in complex fields. 

**Figure 8:**
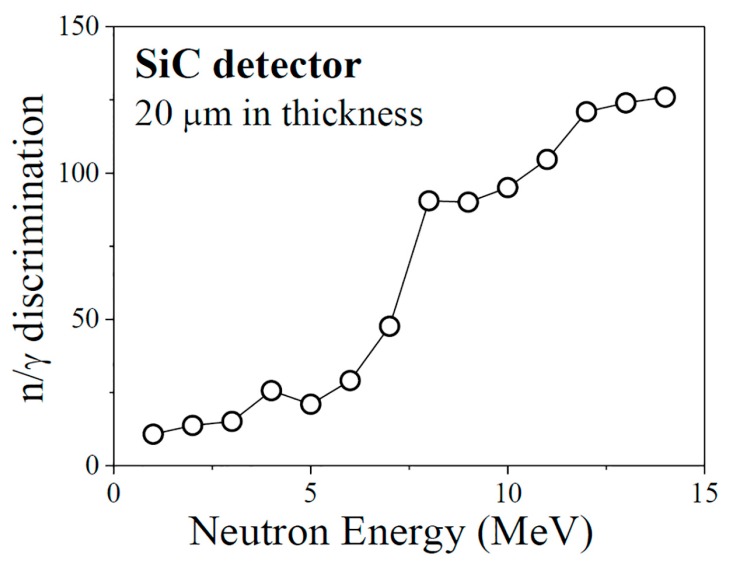
n/γ discrimination of an SiC detector with a thickness of 20 μm, for the neutrons of 14 MeV and the γ-rays of 1.25 MeV.

We studied the n/γ discrimination of the detectors with a thickness of 20 μm and for the neutrons of 14 MeV and γ-rays of 1.25 MeV using MCNP-4C Code [34], and the results are shown in Figure 8. The n/γ discrimination for the neutrons of 14 MeV and the γ-rays of 1.25 MeV is 126, over nine times higher than that of a silicon detector (300 μm in thickness) and seven times higher than that of a diamond detector (300 μm in thickness) according to the results acquired in our former research [35], respectively. In neutron detection, γ-rays always exist in the background. The SiC detector with a thin sensitive volume can attain a low response to background radiation and high n/γ discrimination, and then attain a high signal/noise ratio. As a result, the thin detector shows great advantages in neutron detection in complex radiation fields. 

## 4. Conclusions 

Large-area SiC detectors with a sensitive area of 4 cm^2^ were successfully developed using high-quality epitaxial SiC materials and used in the detection of alpha particles and pulsed X-rays/UV-light. The experiment and simulation indicate that the detectors have a thin sensitive volume, a low dark current, a good energy resolution, and a high n/γ discrimination, though their dimensions are similar with conventional Si detectors. These large-area SiC detectors offer an important option for the detection in large radiation fields, the application of SiC detectors will thus no longer be affected by the limitation of dimensions. With the excellent radiation resistance and outstanding high-temperature endurance, SiC detectors will be more useful in radiation detection in harsh environments and intense radiation fields.
